# Effects of immigrant status on Emergency Room (ER) utilisation by children under age one: a population-based study in the province of Reggio Emilia (Italy)

**DOI:** 10.1186/1472-6963-13-458

**Published:** 2013-11-01

**Authors:** Paola Ballotari, Stefania D’Angelo, Laura Bonvicini, Serena Broccoli, Nicola Caranci, Silvia Candela, Paolo Giorgi Rossi

**Affiliations:** 1 Servizio Interaziendale di Epidemiologia, ASL di Reggio Emilia, Via Amendola 2, Reggio Emilia, Italy; 2 Regional Agency for Health and Social Care Emilia-Romagna Region, Viale Aldo Moro 21, Bologna, Italy

**Keywords:** Emergency, Access, Immigrants, Citizenship, Socioeconomic status, Paediatric care

## Abstract

**Background:**

The primary aim of this study was to assess the effect of immigrant status on Emergency Room (ER) utilisation by children under age one, considering all, non-urgent, very urgent, and followed by hospitalisation visits. The second aim was to investigate the role played by mother’s educational level in the relationship between citizenship and ER utilisation.

**Methods:**

The cohort study included all healthy singleton live births in the years 2008–2009 and residing in the province of Reggio Emilia, followed for the first year of life in order to study their ER visits. The outcomes were the ER utilisation rate for all, non-urgent, very urgent, and followed by hospitalisation visits. The main explanatory variable was mother’s citizenship. Other covariates were mother’s educational level, maternal age, parity, and child gender. Multivariate analyses (negative binomial regression and zero inflated when appropriate) were performed. Adjusted utilisation Rate Ratios (RR) and their 95% Confidence Intervals (95% CI) were calculated. Trend for age in months by citizenship is depicted.

**Results:**

There were 3,191 children (36.4%) with at least one ER visit in the first year of life. Adjusted RR show a significantly greater risk of ER visit for immigrants than for Italians: (RR 1.51; 95% CI 1.39-1.63). Immigrants also had a higher risk of non-urgent visits (RR 1.72; 95% CI 1.48-2.00) and for visits followed by hospitalizations (RR 1.58; 95% CI 1.33-1.89). For very urgent visits, the immigrants had a slightly higher risk compared to Italians (RR 1.25; 95% CI 0.98-1.59).

The risk of ER visits is higher in the first two months of life (RR_1st__vs 3rd-12th_ 2.08; 95% CI 1.93-2.24 and RR _2nd__vs 3rd-12th_ 1.45; 95% CI 1.33-1.58, respectively). Considering all visits, the ER utilisation rate was inversely related with maternal education only for Italians (low educational level 44.0 and high educational level 73.9 for 100 children; p value for trend test < 0.001).

**Conclusions:**

Our study observed a higher use of ER services by immigrant children and, to a lesser extent, by children of less educated Italian mothers. In immigrants, the excess is mostly due to non-urgent visits and only slightly to high acute conditions.

## Background

Emergency Room (ER) is often the only direct access to health care when primary care services are not easily accessible. In many industrialised countries an overuse of ER by immigrants has been observed compared to the native population, both in adults [1-8], and in children [3,5,6]. For other health services, instead, immigrants have lower access than does the native population [8,9].

Some authors focused on higher proportion of non-urgent ER visits [3,4,6], and another study examined immigrants’ reasons for ER access instead of primary health care [10].

Many factors may underlie the differences in ER utilisation, including lack of knowledge about the host country’s health system [1,11], barriers to primary care [1], including language, different habits in the country of origin [2,5], and the level of maternal health literacy. Moreover, some studies found a higher incidence of acute and severe condition requiring ER visits [12,13].

Socioeconomic status (SES), as a social determinant, plays a key role both in health conditions and in health service access [14]. Some studies have demonstrated that disadvantaged groups have higher rates of inappropriate accesses, but also higher rates of underuse of highly recommended procedures or preventive measures [15-17].

Some studies have investigated the combined effect of educational level and immigrant status on birth outcomes [18-20], on hospitalizations of children under age one [20], and on health status [21]. Generally, these studies found a slighter effect of educational level on immigrant population compared to autochthonous population.

In our study we followed a birth cohort of infants through their first birthday to observe their ER access, taking into consideration mother’s citizenship and educational level.

The primary aim of this study was to assess the effect of immigrant status on ER utilisation by children under age one, considering all, non-urgent, very urgent, and followed by hospitalisation visits. The second aim was to investigate the role played by mother’s educational level in the relationship between citizenship and ER utilisation.

## Methods

### Setting and study design

The population of the province of Reggio Emilia, situated in Emilia-Romagna Region, Italy, was 530,343 inhabitants on January 1^st^, 2011 [22]. The foreign population accounted for 13% of the total population (n = 69,060) and the new immigrants born accounted for 26% of the total newborn population. The main countries of origin were Morocco, Albania, India, China, Pakistan, and Romania [23].

Province-wide, there is an Emergency Room Service at the main hospital in the city of Reggio Emilia, as well as Emergency Room Services at the smaller hospitals in the five peripheral health districts. There is no private emergency room service. Emergency Services are available free of charge to children under age 14.

We conducted a population-based cohort study on all the healthy singleton newborns, following them up to age one in order to collect data on all the ER visits.

### Data sources

To identify the study population, we used three different databases.

The *Delivery Assistance Certificates* database (DAC) provides health and sociodemographic information on all live births and stillbirths, sociodemographic information on mother and father, and on pregnancy and delivery.

The *Hospital Discharge* database (HD) contains information about hospitalisation. We used the HD database to retrieve child’s name and surname, date of birth, health status at birth (healthy or unhealthy, identified according to Emilia-Romagna Region criteria ^a^), and gender.

The *Emergency Room* database (ER) contains information on all visits to ER services in the province of Reggio Emilia, including the level of urgency and the modality of discharge.

### Population inclusion criteria

**Figure 1:**
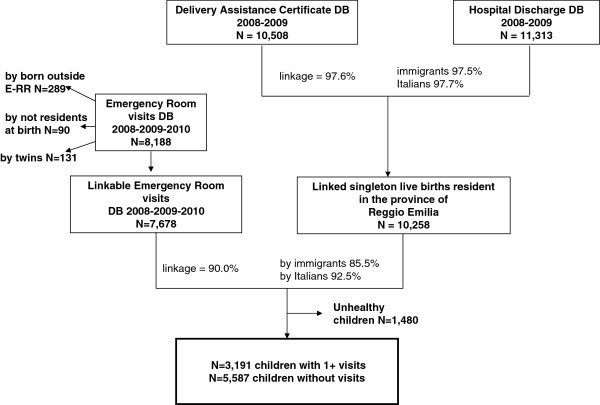
Record linkage process, flow chart, Reggio Emilia (Italy), 2008–2010.

The study population consisted of all healthy singleton live births in Emilia-Romagna Region in the years 2008 and 2009, resident in the province of Reggio Emilia. The study database was obtained by record linkage between the three above-mentioned databases (Figure 1).

The 2008–2009 DAC database was used to identify all singleton live births in Emilia-Romagna Region resident in the province of Reggio Emilia (n = 10,508). The database was linked to the 2008–2009 HD database; from this, a cohort of 10,258 children was obtained (success of record linkage: 97.6%, with no difference between immigrants and Italians).

The new cohort was linked with eligible records of ER database (n = 7678).

Failed links were due to misspelling of name and/or surname, mistakes in residence assignment, because children were born at home, or because the DAC was not filled out at delivery [24]. Finally, the analysis was restricted to healthy children as defined according to E-RR criteria^a^. The final cohort included 8,778 children, 3,191 of whom had at least one visit and 5,587 had no visit.

### Outcomes and other variables

The main outcome was the ER utilisation rate, considering all visits. In addition, the ER utilisation rates for non-urgent conditions, very urgent visits, and visits followed by hospitalisation were analysed. To define “non-urgent conditions” and “very urgent visits” we used the triage code category, which indicates the level of urgency: red (emergency, failure of vital signs); yellow (urgency with no immediate life-threatening condition) - both considered as “very urgent visits”-, green (minor urgency), white (non-urgent condition). This last category includes cases not appropriate for the ER and is used when the patient could have referred to general practitioner or to other primary care services [25]. The use of triage code in epidemiological studies has been already validated [26].

The main explanatory variable was the maternal citizenship. All mothers who were autochthonous citizens and all immigrants from Highly Developed Countries (HDC) were grouped as Italians, while mothers who were citizens of High Migration Countries were considered immigrants (HMC) [27]. In the geographical analysis, the HMC were grouped according to WHO areas.

Other covariates included in the models were educational level, classified as high (i.e. university degree; schooling years (SY) ≥ 16), medium (i.e. secondary level; 13 ≤ SY < 16), or low level (SY < 13); maternal age (<25, 25–34, > = 35 years of age); parity (no previous live birth, at least one previous live birth); child gender (male or female).

### Statistical analyses

We summarize our data using percentages to describe characteristics of the cohort, number of visits per child, type of visit according to triage category, and type of discharge.

In our study we defined the ER utilisation rate as the ratio between the total number of ER visits carried out by the children included in the cohort and the total population in the cohort, multiplied by 100 (all the children were followed up for one year from their birth).

Utilisation rates by citizenship and age in months are presented; rate ratios comparing the first month and the second month of life to months from third to twelfth are reported with their relative 95% Confidence Intervals (95% CI).

To determine the crude and adjusted rate ratios, negative binomial regression models were used instead of Poisson regression because assumption of conditional mean is equal to conditional variance was violated, i.e., the overdispersion parameter was significantly different from zero [28]. For non-urgent visits, we used a zero-inflated negative binomial regression, because the number of zeroes turns out to be inflated [28]. The likelihood ratio test of alpha = 0 was used to compare the negative binomial regression model to Poisson regression, while the Vuong test was used to compare the zero-inflated negative binomial to the standard negative binomial model [29]. Covariates described below were considered in multivariates models.

Tests for linear trend on the effect of maternal educational level were calculated. To test the interaction between maternal citizenship and educational level a Wald test was applied. Predicted utilisation rates and probabilities of at least one visit by citizenship and educational level were calculated.

For the “visits followed by hospitalisation” we performed a further analysis using visits as statistical units and hospitalisations as outcome, i.e. modelling the probability of being hospitalised given that a ER visit occurred, and applying a multivariate logistic regression with the triage category as additional predictor.

For all analyses, a 5% significance level was used. Data analysis was performed using Stata/IC 11.0.

### Ethical approval

This is an observational study and data were collected retrospectively. The Local Health Authority of Reggio Emilia was responsible for collecting and elaborating these data. The study was commissioned by the Local and Regional Health Authorities. No ethical approval was required according to Italian law 211/2003 which explain that no ethic committee’s permission is required for this kind of studies. According to Italian privacy law, no patients’ or parents’ consent is required for large retrospective population-based studies and if data are published only in aggregated form.

## Results

### Description of the cohort

The studied cohort included 8,778 healthy children, 2,383 of whom were immigrants (27.1%). Compared to the Italians, immigrant mothers were younger, had a lower educational level, and more frequently had at least one previous birth (Table 1).

**Table 1:** Characteristics of cohort by citizenship, Reggio Emilia (Italy), 2008-2010

	**Italians**		**Immigrants**		**Whole Cohort**	
**Characteristics**	**N**	**%**	**N**	**%**	**N**	**%**
**Child gender**						
Male	3,237	50.6	1,218	51.1	4,455	50.8
Female	3,158	49.4	1,165	48.9	4,323	49.2
**Maternal age**						
<25 years	192	3.0	294	12.3	486	5.5
25-34 years	1,755	27.4	788	33.1	2,543	29.0
> = 35 years	4,448	69.6	1,301	54.6	5,749	65.5
**Mother’s educational level**						
High	1,517	23.7	230	9.7	1,747	19.9
Medium	3,228	50.5	734	30.8	3,962	45.1
Low	1,650	25.8	1,419	59.5	3,069	35.0
**Previous live births**						
No	3,277	51.2	992	41.6	4,269	48.6
Yes	3,118	48.8	1,391	58.4	4,509	51.4
TOTAL	6,395	100.0	2,383	100.0	8,778	100.0

### ER utilisation

As shown in Table 2, 36.3% of children had at least one visit to the ER. The proportion was higher for immigrant children than for Italian children; the utilisation rate for the whole cohort was 64.1 (5,630/8,778 × 100 children), and the rate was higher for immigrant than for Italians: 90.6 (2,158/2,383 × 100 children) and 54.3 (3,472/6,395 × 100 children), respectively.

**Table 2:** Visits frequency per child and type of visits by citizenship, Reggio Emilia (Italy), 2008-2010

	**Italians**		**Immigrants**		**Total**	
**N. of visits per child**^**§ **^**°**	**N**	**%**	**N**	**%**	**N**	**%**
0 visits	4,291	67.1	1,296	54.4	5,587	63.7
1 or more visits	2,104	32.9	1,087	45.6	3,191	36.3
1	1,311	20.5	540	22.7	1,851	21.1
2	482	7.5	277	11.6	759	8.7
3	177	2.8	142	6.0	319	3.6
4	79	1.2	69	2.9	148	1.7
5	24	0.4	28	1.2	52	0.6
6+	31	0.5	31	1.3	62	0.7
Type of visit *						
*Total visits*	*3,472*	*100.0*	*2,158*	*100.0*	*5,630*	*100.0*
*Triage code*						
*Red/yellow*	*232*	*6.7*	*129*	*6.0*	*361*	*6.4*
*Green*	*2,450*	*70.6*	*1,392*	*64.5*	*3,842*	*68.2*
*White*	*790*	*22.7*	*637*	*29.5*	*1,427*	*25.4*
Type of discharge *						
*Visits followed by hospitalisation*	*427*	*12.3*	*288*	*13.4*	*715*	*12.7*

^§^ Analysis units are children.

^*^ Analysis units are visits.

° overdispersion test (i.e. Likelihood-ratio test of alpha = 0): p value < 0.001 for total cohort, for Italians and for immigrants.

Of 5630 accesses, 12.7% ended with hospitalisation (Table 2). Yellow and red triage codes accounted for 6.4% of the accesses, while white triage code was 25.4%. The remaining 68.2% was green triage code, i.e. minor urgency that can be delayed without serious consequences.

**Figure 2:**
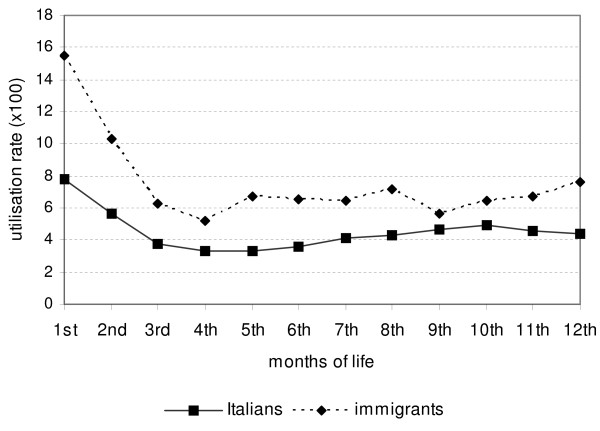
ER utilisation rate (per 100 children) by citizenship and age in months, Reggio Emilia (Italy), 2008–2010.

The ER utilisation rate for immigrant children was higher than for Italians at any age (in months) (Figure 2). Moreover, the risk of ER access was higher in the first and second months of life: RR_1st__vs 3rd-12th_ 2.08 (95% CI 1.93-2.24) and RR_2nd__vs 3rd-12th_ 1.45 (95% CI 1.33-1.58), respectively.

The risk excess for immigrants remained after adjusting for other covariates (Table 3). The analyses by mother’s geographical area of origin showed an increased risk of ER visits for all areas (compared to Italian mothers); the risk was double for Sub-Saharan, and only slightly higher for Asia. The lower maternal educational level increased the risk for ER visit, with a significant trend (p < 0.001). Female gender and not firstborn consistently resulted in a lower ER visit risk, while the mother’s age at delivery did not lead to conclusive evidence.

**Table 3:** ER visits: crude and adjusted utilisation Rate Ratio (RR) and 95% Confidence Interval (95% CI), Reggio Emilia (Italy), 2008-2010

**Characteristics**	**Crude RR**	**95% CI**	Adjusted RR*	**95% CI**
**Citizenship**				
Italian	1.00	-	1.00	-
Immigrant^	1.67	1.54-1.80	1.51	1.39-1.63
***Geographical Area***				
*Central and Eastern Europe (n = 639)*	*1.74*	*1.52-1.98*	*1.55*	*1.36-1.77*
*Asia (n = 695)*	*1.26*	*1.11-1.44*	*1.10*	*0.96-1.25*
*Northern Africa (n = 735)*	*1.77*	*1.57-2.00*	*1.60*	*1.42-1.81*
*Sub-Saharan Africa (n = 223)*	*2.31*	*1.89-2.83*	*2.12*	*1.75-2.59*
*Latin America (n = 91)*	*1.86*	*1.34-2.59*	*1.86*	*1.36-2.54*
**Mother’s educational level**				
high	1.00	-	1.00	-
medium	1.13	1.02-1.25	1.09	0.99-1.21
low	1.71	1.54-1.90	1.51	1.36-1.68
*trend*	*p*^*§*^ *< 0.001*		*p*^*§*^ *< 0.001*	
**Age at delivery**				
age <25	1.61	1.38-1.88	1.23	1.06-1.44
age 25-34	1.00	-	1.00	-
age > =35	0.98	0.90-1.06	1.03	0.95-1.11
**Child gender**				
Male	1.00	-	1.00	-
Female	0.88	0.82-0.95	0.89	0.82-0.95
**Previous live birth**				
None	1.00	-	1.00	-
At least one	0.86	0.80-0.92	0.80	0.75-0.87
*Likelihood-ratio test for alpha = 0*	-	-	*p < 0.001*	
*Vuong test*			*p = 0.099*	

* Adjusted utilisation Rate Ratio (RR) and 95% Confidence Intervals (95% CI) were obtained using multivariate negative binomial regression model with citizenship, mother’s educational level, age at delivery, child gender, and previous live birth as covariates.

^ Immigrants from HMC were classified by geographical area according to WHO criteria. There were no children whose mother was of Western Pacific origin.

*§*p value for linear trend test.

The model showed a significant over-dispersion, i.e. children who already had a visit were more likely to have further visits.

### Analysis of appropriateness and urgency

The type of access for which immigrants had the strongest relative risk was white triage, i.e., non-urgent visits (RR 1.72; 95% CI 1.48-2.00), (Table 4). Immigrants had also a slightly higher risk than did Italians of higher urgency accesses, i.e. red/yellow triage codes (RR 1.25; 95% CI 0.98-1.59). Finally, immigrants also had a higher risk of ER visits followed by hospitalisation (RR 1.58; 95% CI 1.33-1.89). Performing multivariate logistic regression of the probability of being hospitalised after ER visit, immigrants were more likely than Italians to end ER visit with an in-hospital admission, OR 1.21 (95% CI 1.01-1.45), even with the same level of urgency.

**Table 4:** Adjusted utilisation Rate Ratio (RR), and 95% Confidence Interval (95% CI) for ER visits with white, red/yellow triage code and followed by hospitalisation, Reggio Emilia (Italy), 2008-2010

	**White triage code^**		Red/yellow triage codes*		Followed by hospitalisation*	
**Characteristics**	**RR**	**95% CI**	**RR**	**95% CI**	**RR**	**95% CI**
**Citizenship**						
Italian	1.00	-	1.00	-	1.00	-
Immigrant	1.72	1.48-2.00	1.25	0.98-1.59	1.58	1.33-1.89
**Mother’s educational level**						
high	1.00	-	1.00	-	1.00	-
medium	0.94	0.79-1.13	1.12	0.80-1.57	1.35	1.05-1.72
low	1.56	1.28-1.90	1.75	1.25-2.46	1.58	1.22-2.04
*trend*	*p*^*§*^ *< 0.001*		*p*^*§*^ *< 0.001*		*p*^*§*^ *= 0.001*	
**Age at delivery**						
age <25	1.33	1.04-1.71	1.33	0.84-2.12	1.09	0.78-1.53
age 25-34	1.00	-	1.00	-	1.00	-
age > =35	1.14	0.97-1.33	1.18	0.92-1.52	0.85	0.72-1.02
**Child gender**						
Male	1.00	-	1.00	-	1.00	-
Female	0.90	0.79-1.03	0.72	0.57-0.89	0.88	0.75-1.03
**Previous live birth**						
None	1.00	-	1.00	-	1.00	-
At least one	0.83	0.70-0.98	1.12	0.90-1.40	1.14	0.97-1.35
*Likelihood-ratio test for alpha = 0*	*p < 0.001*		*p < 0.001*		*p < 0.001*	
*Vuong test*	*p = 0.032*		*p = 0.154*		*p = 0.080*	

^ Adjusted utilisation Rate Ratio (RR) and 95% Confidence Intervals (95% CI) were obtained using multivariate zero inflated negative binomial regression model with citizenship, mother’s educational level, age at delivery, child gender, and previous live birth as covariates.

* Adjusted utilisation Rate Ratio (RR) and 95% Confidence Intervals (95% CI) were obtained using multivariate negative binomial regression model with citizenship, mother’s educational level, age at delivery, child gender, and previous live birth as covariates.

*§*p value for linear trend test.

Child gender differences persisted for the three outcomes analysed. For previous live births, the results for non-urgent visits agreed with those found for all visits; for the other two outcomes, having had a previous child did not diminish the probability of an access.

All the outcomes showed an over-dispersion; non-urgent visits also revealed an inflation of children without this kind of visit.

### Effect of mother’s educational level

**Figure 3:**
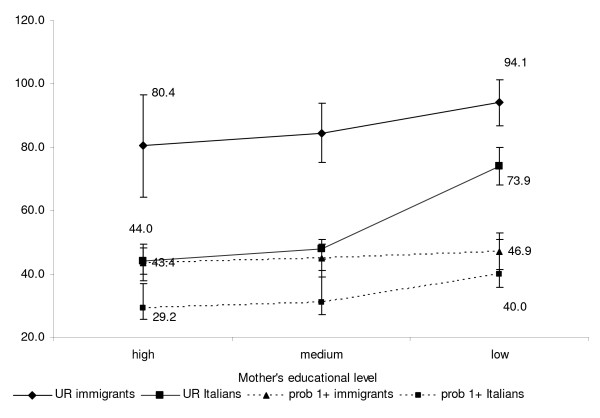
**Estimated ER utilisation rate (per 100 children) and estimated probability of at least one visit (per 100 children) by educational level and citizenship, Reggio Emilia (Italy), 2008–2010.** Utilization rates and probabilities are predicted using multivariate negative binomial regression model stratified by citizenship and adjusted for age at delivery, child gender, and previous live birth.

Considering all visits, the effect of mother’s education level differed between Italians and immigrants, (test for interaction p = 0.0001). This was evident when considering both the predicted ER utilisation rate (per 100 children) and the predicted probability of at least one visit (per 100 children) by educational level and citizenship, keeping all other variables at their means (Figure 3), even if the Italian mother’s educational level had a stronger impact on ER utilisation rate than on the probability of at least one access.

The modification effect of mother’s educational level was present also for non-urgent visits (test for interaction p = 0.0002), whereas disappeared for very urgent visits (p = 0.8698) and for visits followed by hospitalisation (p = 0.1150).

## Discussion

### Use of ER services

Our population-based study confirms a higher use of ER services in the first year of life by children with immigrant mothers compared to those with Italian mothers [3,5,6,11]. The first two months of life are those at higher risk for all children.

For every mother’s geographical area of origin, the study detected a greater risk for immigrant children compared to Italians, more evident for children whose mother came from Sub-Saharan Africa. Norredam [1] found higher risk for ER utilisation for adults born in Somalia, Turkey, and ex-Yugoslavia. Rué [2] detected the highest risk for adult women from the Maghreb and Sub-Saharan Africa, and Bonvicini [3] found the highest risk for adult Moroccan and Tunisian immigrants.

In our cohort those who had at least one access were more likely to have other accesses. Despite the fact that we included only healthy newborns at delivery discharge, the cohort surely included some children with chronic pathologies not diagnosed at birth that could explain the presence of a group at higher risk of repeated visits [30]. Furthermore, behavioural and cultural factors may define a group of “frequent users” [31].

To investigate whether overall increased risk was related to a higher incidence of acute conditions requiring emergency visits or to behavioural factors influencing the use of health services, we split the outcome according to urgency and severity of the conditions determining the visit. For very urgent visits, which should reflect higher incidence of acute conditions, the immigrant population had a slightly higher risk compared to Italians.

For visits followed by hospitalisation the risk was higher for immigrants than for Italian. This type of access could include more severe and complex cases as well as those cases where the healthcare personnel’s confidence in caregiver compliance to manage follow up at home was low. Our data show that the probability of being hospitalised after ER visit is only slightly higher in immigrants than in Italians. An excess of hospitalisation cannot thus be explained entirely by healthcare personnel’s behaviours.

A higher incidence of acute condition in immigrants is consistent with the results of a previous study conducted in the ER services of the Lazio Region (Italy): when analysing only traumas, for which non-urgent access is by definition quite rare, the immigrant population had a higher proportion of very urgent accesses [12]. Further, a study conducted in Switzerland [13] found that children with an immigrant background were overrepresented in Paediatric Intensive Care Units (PICU).

The risk for immigrants to have an inappropriate ER visit is more than double that of Italians. This finding agrees with other studies [3,4,32,33] except one [34], which did not find any differences between immigrants and Italians in terms of non-urgent visits.

It is worth noting that the model for white triage codes is the only one with a significant zero inflated component. A possible explanation is that the population not having white code accesses is composed of two groups: 1) healthy children and 2) those who did not go to the ER when experiencing a non-urgent condition, i.e. they either accessed primary care or did not receive medical care.

In conclusion, both higher incidence of acute conditions and higher inappropriate use are present in immigrants, with the impact of the second more relevant, in term of number of visits, than the first one.

The high use of ER services might be explained by the fact that the ER is the easiest and most immediate point of access to healthcare. Indeed, access to primary care might present several linguistic/logistic barriers that could result in more frequent, and inappropriate, use of the emergency services [10,32,33]. Kubicek [35] highlights the limited ability of caregivers to accurately judge the urgency of the presenting conditions. Further, it is possible that immigrant women use the hospital as first healthcare access point because the hospital is often the only healthcare facility in their home country.

### The effect of educational level

Our results show that the effects of education differ according to citizenship: among Italians, education level is inversely associated with ER utilisation rate; among immigrants, no relation between these variables is detected. For women with the same education level, immigrants have a higher risk of visits compared to Italians, although this difference decreases at lower education levels. The difference is more marked when considering utilisation rates instead of probability of at least one visit. We found this effect modification for all visits and non-urgent visits but not for the remaining outcomes.

Regarding low educational level and low socioeconomic status, Spencer [36] found higher risk for multiple hospital admissions in children from deprived areas, as did Braun [37] for ER utilisation by populations with lower socioeconomic status. Thrane [38] found a higher risk of hospitalisation for infectious diseases in children whose mothers had only basic schooling.

### Limits

The only component of socioeconomic status (SES) that we could measure was educational level. Mother’s educational level is considered a good proxy of SES [39] and is strongly related to several health outcomes, including birth outcomes [40,41]. Unfortunately, information on educational level is collected using the highest degree obtained and great diversity in the education systems in the maternal countries of origin could determine a non homogeneous classification [39]; to have a single classification we tried to translate diplomas/ degrees into schooling years but the translation introduced a strong approximation.

Further, it was not possible to ascertain how long the mothers had been living in Italy before delivery since this information is not recorded in any health administrative database: length of stay may measure the integration process and reduce the difference in the pattern, as reported by another study [34].

Another limitation of the study was the shortage of clinical information available on emergency visits, making it impossible to conduct analyses by admission problem self-reported and/or by diagnosis.

Finally, the record linkage between the ER visits and the newborn cohort was more accurate for Italians than for immigrants (this is typically due to misspelling of names). As a result, we may have slightly underestimated the excess of risk in immigrants.

## Conclusions

Our study observed a higher use of ER services by immigrant children and, to a lesser extent, by children of less educated Italian mothers. In immigrants, the excess was mostly due to non-urgent visits and only slightly to urgent and severe conditions.

The risk of ER visits is higher in the first two months of life.

Based on this epidemiological evidence, it appears important to plan a public health intervention targeting foreign and low education level Italian mothers in the first days after delivery, as supported by international literature [42-44] and by recent Italian experience [45].

## Endnotes

^a^Emilia-Romagna Region healthy child definition: discharge by nursery and not transferred to other hospital and birth date = admission date and mode of discharge other than “dead” or “transferred”.

## Abbreviations

CI: Confidence intervals; DAC: Delivery assistance certificates (database); ER: Emergency room; E-RR: Emilia-Romagna Region; HD: Hospital discharge (database); HDC: Highly developed countries; HMC: High migration countries; RR: Utilisation rate ratio; SES: Socio-economic status; SY: Schooling years; PICU: Paediatric intensive care units.

## Competing interests

The authors declare they have no competing interests.

## Authors’ contributions

All the authors have contributed to this study. SC conceived the study and reviewed the manuscript. SD, PB, and LB performed the main statistical analyses, interpreted the results, and drafted the manuscript. PGR helped to define statistical analyses and reviewed the manuscript. NC and SB reviewed the manuscript. All authors have read and approved the final manuscript.

## Pre-publication history

The pre-publication history for this paper can be accessed here:

http://www.biomedcentral.com/1472-6963/13/458/prepub
